# Helping someone with problem drug use: a delphi consensus study of consumers, carers, and clinicians

**DOI:** 10.1186/1471-244X-11-3

**Published:** 2011-01-05

**Authors:** Anna H Kingston, Amy J Morgan, Anthony F Jorm, Kate Hall, Laura M Hart, Claire M Kelly, Dan I Lubman

**Affiliations:** 1 Orygen Youth Health Research Centre, Centre for Youth Mental Health, University of Melbourne, Victoria, Australia; 2 Turning Point Alcohol and Drug Centre, Eastern Health and Monash University, Victoria, Australia

## Abstract

**Background:**

Problem use of illicit drugs (i.e. drug abuse or dependence) is associated with considerable health and social harms, highlighting the need for early intervention and engagement with health services. Family members, friends and colleagues play an important role in supporting and assisting individuals with problem drug use to seek professional help, however there are conflicting views about how and when such support should be offered. This paper reports on the development of mental health first aid guidelines for problem drug use in adults, to help inform community members on how to assist someone developing problem drug use or experiencing a drug-related crisis.

**Methods:**

A systematic review of the scientific and lay literature was conducted to develop a 228-item survey containing potential first-aid strategies to help someone developing a drug problem or experiencing a drug-related crisis. Three panels of experts (29 consumers, 31 carers and 27 clinicians) were recruited from Australia, Canada, New Zealand, the United Kingdom, and the United States. Panel members independently rated the items over three rounds, with strategies reaching consensus on importance written into the guidelines.

**Results:**

The overall response rate across three rounds was 80% (86% consumers, 81% carers, 74% clinicians). 140 first aid strategies were endorsed as essential or important by 80% or more of panel members. The endorsed strategies provide information and advice on what is problem drug use and its consequences, how to approach a person about their problem drug use, tips for effective communication, what to do if the person is unwilling to change their drug use, what to do if the person does (or does not) want professional help, what are drug-affected states and how to deal with them, how to deal with adverse reactions leading to a medical emergency, and what to do if the person is aggressive.

**Conclusions:**

The guidelines provide a consensus-based resource for community members who want to help someone with a drug problem. It is hoped that the guidelines will lead to better support and understanding for those with problem drug use and facilitate engagement with professional help.

## Background

The Mental Health First Aid (MHFA) program is an educational course designed to teach members of the public skills in recognizing and responding to mental disorders in another person [1]. MHFA is modelled on physical first aid, and is the early help provided to someone developing a mental disorder, as well as assistance during mental health crisis situations. The MHFA program teaches first aid for a variety of mental health problems, including depression, anxiety, trauma, psychosis, eating disorders and suicidal behaviour. The program was developed in response to the often poor mental health literacy of members of the public, who may lack knowledge about mental disorders and how they can best be treated, The MHFA program has separate versions for adults and adults assisting youth, and has been adapted for Indigenous Australians and some non-English speaking immigrant groups. Controlled trials have shown that the program improves recognition of mental disorders, beliefs about treatments, and helping behaviour provided, as well as reducing social distance [2]. More than 100,000 people have completed a MHFA course in Australia and the course has spread to 14 other countries.

The MHFA program also teaches participants how to assist a person who has problem use of illicit drugs. Within the program, first aid for problem drug use is defined as the help provided to a person developing a drug use problem or experiencing a drug-related crisis (e.g. overdose, drug-induced psychosis). The first aid is given until appropriate professional treatment is received or until the crisis resolves. Drug use disorders are associated with substantial morbidity and mortality, with significant impacts evident on the user, their family, and the broader community. Despite such harms, many individuals with drug use disorders do not seek treatment, with delays in accessing professional help often for a decade or more [3,4]. Nevertheless, community members and concerned others (family, friends, colleagues) have an important role in supporting and assisting a person with a drug use disorder to seek treatment or change their behaviour [5]. However, there are conflicting views about how to support a person with illicit drug problems, with some people believing that a person cannot be helped until they have 'hit bottom', (i.e. their drug use causes overwhelming problems in multiple areas of their life) [6]. The MHFA program can thus give members of the public confidence in providing support and assistance to someone who is developing a drug use problem.

To increase the evidence base of MHFA, guidelines for mental health first aid strategies have been developed using expert consensus (the Delphi method). Evidence from expert consensus is particularly suited for this type of intervention, as it is not feasible to use the gold-standard randomised controlled trial to investigate the effectiveness of different first aid strategies for developing mental disorders. Consensus from consumers and carers in addition to consensus from clinicians is important because consumers and carers have different perspectives and types of experience to draw on, and they represent people who might typically receive or give first aid. Guidelines have been developed for a number of developing mental health problems and crises [7-13], including problem alcohol use [14].

This paper reports on the development of mental health first aid guidelines for problem drug use in adults. We defined problem drug use as using cannabis, ecstasy, amphetamines (including methamphetamine), cocaine or heroin, at levels that are associated with both short- and long-term harm. Problem drug use therefore includes drug-affected states, drug abuse and drug dependence. The aim was to get consensus between experts on the best way a member of the public could help someone who was developing problem drug use, or who was experiencing a drug-related crisis. Once established, these guidelines would inform an update of the MHFA training program, and would empower members of the public to provide crucial and appropriate support to family, friends or loved ones experiencing or developing a drug use problem.

## Methods

### The Delphi Method

The Delphi method involves a panel of experts making private, independent ratings of agreement with a series of statements [15]. Statements about mental health first aid strategies for problem drug use were derived from a search of the lay and scientific literature, and these were presented to a panel of experts in three sequential rounds. New strategies suggested by panel members were included as statements in the second round for all experts to rate. A summary of group ratings was fed back to the panel members after the first and second rounds. Panel members could choose to either change or maintain their original ratings, in light of the group ratings. This process resulted in a list of statements that had substantial consensus in ratings, and statements with low or conflicting ratings were discarded.

### Panel formation

Consumers, carers and clinicians from Australia, Canada, New Zealand, the United Kingdom and the United States were recruited into three separate panels, all with expertise in problem drug use. Consumers (people with a past history of problem drug use) and carers (people with experience caring for someone with problem drug use) were recruited by distributing information about the study to consumer and carer organizations associated with substance use or mental health issues in each country. We specified that any consumers and carers who took part needed to be in an advocacy role, which we defined as having represented the interests of drug users or their carers within the community. This was to ensure that participants had an understanding of problem use beyond their personal experience. Consumers and carers were offered a bookshop voucher worth AUD 33 for each round of the survey they completed. More than 300 clinical experts were initially invited to participate, sourced from the editorial boards of 8 leading peer-reviewed substance use journals, addiction specialist colleges and societies, or experienced clinicians working within alcohol and other drug settings. A strength of the Delphi method is that it does not require representative sampling; it requires panel members who are information- and experience-rich.

Eighty-seven panel members from Australia, Canada, New Zealand, the United Kingdom and the United States were recruited. There were 29 consumers, 31 carers, and 27 clinicians. Fifty-one panel members were female (59% of the consumers, 74% carers, 33% of the clinicians). The median age category was 40-49 years for the consumers, 50-59 years for the carers and 50-59 years for the clinicians. Out of the 27 clinicians on the panel, there were 8 psychologists, 5 psychiatrists, 3 medical specialists, 1 pharmacist, 1 psychopharmacologist, 1 registered nurse, 1 dual diagnosis clinician, 1 ACT (Acceptance and Commitment Therapy) therapist, 5 professors, 1 research fellow, 6 researchers, 1 consultant, and 1 director of a specialist centre (figures do not add up to 27 as clinicians reported multiple roles).

### Questionnaire development and administration

A systematic literature review was conducted of websites, books and journal articles for strategies about how to help someone who may be developing or experiencing a drug use problem. This involved a comprehensive Internet search in google search engines [16-18]. The following search terms were entered into each: *cannabis *or *ecstasy *or *amphetamine *or *cocaine *or *opioids*. The first 50 sites for each set of search terms were examined for statements about how to help someone who may have a drug use problem. Any links that appeared on these web pages that the authors thought may contain useful information were followed. Relevant journal articles were located on PsycINFO and PubMed. The 50 most popular books on the Amazon website were also selected and reviewed. We obtained suggestions for first aid strategies from approximately 18 websites, 7 books, 1 pamphlet and 2 journal articles. The majority of first aid strategies came from websites, as few books and journal articles focused on pre-clinical interventions. In addition, the questionnaire content was informed by a questionnaire previously developed to create first aid guidelines for problem drinking [14], as well as strategies suggested by the working group to fill perceived gaps in the questionnaire's content.

The information gathered from these sources was analysed by one of the authors (AK) and written up as individual survey items. This document was presented to a working group, who screened the items to ensure they fitted the definition of first aid for problem drug use, were comprehensible, and had a consistent format, while remaining as faithful as possible to the original wording of the information. After several draft surveys, the group produced a list of 228 items that formed the first survey sent to panel members. The Round 1 survey was organized into 13 sections (see Table 1). Panel members were asked to rate the importance of each item as a first aid strategy, bearing in mind that a first aider was a member of the general public and therefore did not necessarily have a medical or clinical background. The rating scale used was essential, important, depends, unimportant, should not be included, don't know. The Round 1 survey also included comment boxes that allowed panel members to give feedback after each section. To analyse the comments that panel members had written in the first round questionnaire, one of the authors (AK) read through all the comments and wrote them up as draft first aid strategies. The working group evaluated the suggested draft strategies to determine whether they were original ideas that had not been included in the first round questionnaire. Any strategy that was judged by the group to be an original idea was included as a new item to be rated in the second round questionnaire.

**Table 1:** Round 1 survey sections and number of items

*Section*	*Number of items*
Understanding problem drug use	13
Recognising problem drug use	7
Approaching the person about their problem drug use	68
Information and support for the person who wants to stop using drugs	6
Professional help	25
What to do if the person is unwilling to change their drug use	15
Understanding drug-affected states	7
Interacting with, and responding to, the drug-affected person	7
Maintaining the drug-affected person's safety	9
General principles for recognising and responding to adverse reactions	9
Responding to particular adverse reactions	15
Responding to medical emergencies	15
What to do if the person is agitated or aggressive	32

Panel members completed the questionnaires online (using SurveyMonkey [19]). The study was approved by the Human Research Ethics Committee at the University of Melbourne.

### Statistical analysis

On completion of each round, the survey responses were analysed by obtaining percentages for the consumer, carer and clinician panels for each item. The following cut-off points were used:

#### Criteria for accepting an item

• If at least 80% of the consumer, carer and clinician panels rated an item as essential or important as a first aid guideline for problem drug use, it was included in the guidelines.

#### Criteria for re-rating an item

• If 80% or more of the panel members in one group rated an item as essential or important as a first aid guideline for problem drug use, we asked all panel members to rerate that item in the next round.

• If 70-79% of panel members from all three groups rated an item as essential or important, we asked all panel members to rerate that item.

• Items were re-rated once only. If an item was not endorsed after two rounds it was excluded from the guidelines.

#### Criteria for rejecting an item

• Any items that did not meet the above conditions were excluded.

## Results

**Figure 1:**
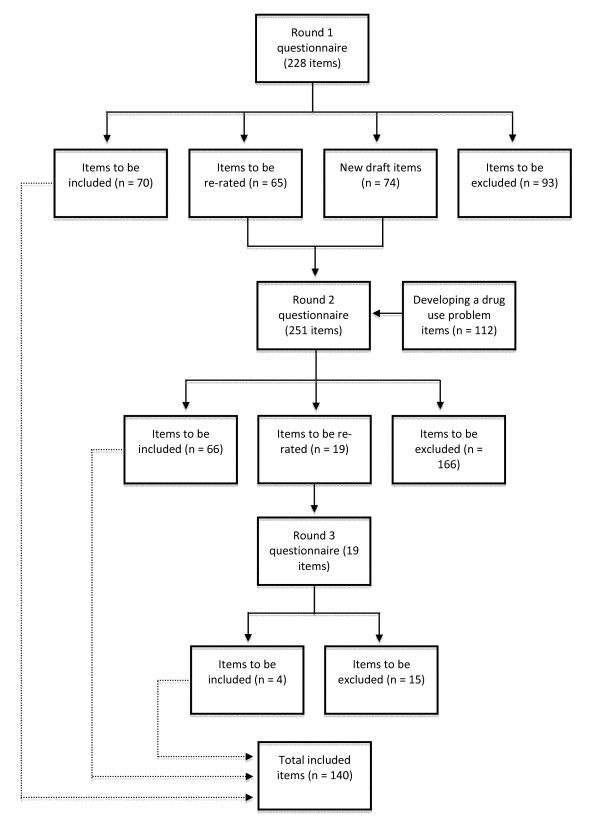
**Overview of items included, excluded, created and re-rated in each round of the survey**.

See Figure 1 for an overview of the numbers of items that were included, excluded, created and re-rated in each round of the survey. The response rate of those who took part in all three rounds was 80% (86% consumers, 81% carers, 74% clinicians). See Table 2 for the number of panel members who completed each round.

**Table 2:** Participant numbers for each round of the survey

	*Round 1* n	*Round 2* n (%)	*Round 3* n (%)
Consumer	29	26 (90%)	25 (86%)
Carers	31	25 (81%)	25 (81%)
Clinicians	27	23 (85%)	20 (74%)

Total	87	74 (85%)	70 (80%)

Participant feedback from the Round 1 questionnaire indicated there was confusion about the difference between *physical *first aid and *mental health *first aid. In addition, it appeared that the panel members assumed that the items in the Round 1 questionnaire were targeting people with entrenched drug disorders, rather than those who were developing drug use problems. To address these concerns, the Round 2 questionnaire clearly stated the distinction between physical first aid and mental health first aid. In addition, a new section was added called 'Developing a drug use problem'. In this section, all items from the Round 1 questionnaire sections about 'approaching the person', 'information and support for stopping or reducing drug use' and 'professional help' were resubmitted to the panel. These sections were prefaced with the statement, "these items are about helping a person who is just starting to develop problems as a result of their drug use." Thus, a 251-item survey was developed for Round 2. It comprised the 74 new items suggested by panel members, the 65 items from round 1 to be re-rated in Round 2, and 112 items in the 'Developing a drug use problem' section. However, analysis showed that presenting items from the Round 1 questionnaire in the new section 'Developing a drug use problem' made little difference to which items were endorsed. Consequently, the section on 'Developing a drug use problem' was not submitted for re-rating in Round 3, nor was the distinction between 'entrenched' and 'developing' drug use problems included in the guidelines.

Across the three rounds, 140 strategies were rated as essential or important by ≥80% of the panel members (see Additional File 1: Items that received 80% consensus across both the consumer, carer and clinician panels). Overall, ratings of whether items were essential or important were similar across the consumer, clinician, and carer panels, with correlations of r = 0.89 between consumers and carers, r = 0.88 between consumers and clinicians, and r = 0.89 between and clinicians and carers. One of the authors (AK) prepared the final guidelines by grouping items of similar content under specific headings. The items were strung together into prose so that the guidelines offered the first aider a coherent approach to first aid for problem drug use. The guidelines retained the original wording of the items as much as possible, whilst remaining easy-to-read. Furthermore, some items were given examples to clarify the general nature of the advice, for instance, including some examples of consequences of problem drug use. Additional items about 'what to do if the person is aggressive' were included in these guidelines. These items were taken from the first aid guidelines for problem drinking, which were developed using the same Delphi process used to develop the current guidelines [14]. The guidelines were then given to panel members for final comment, feedback and endorsement. Any comments made by panel members were presented to the working group and integrated into the document if they made the text clearer. However, new content was not accepted at this stage.

The final guidelines (see Additional File 2) provide information and advice on what is problem drug use and its consequences, how to approach the person about their problem drug use, tips for effective communication, what to do if the person is unwilling to change their drug use, what to do if the person does (or does not) want professional help, what are drug-affected states and how to deal with them, how to deal with adverse reactions leading to a medical emergency, and what to do if the person is aggressive.

## Discussion

This research aimed to identify first aid strategies that members of the public could carry out to assist someone developing problem drug use or experiencing drug-related crises. We have shown that it is possible for experts to reach consensus on first aid for problem drug use that can be applied across several types of illicit drugs. Over one-hundred first aid strategies were endorsed from a comprehensive range of first aid suggestions. The endorsed strategies were written into a cohesive guideline document. This is currently the only resource that provides consensus on mental health first aid strategies for problem drug use between expert clinicians, carers, and consumers.

Although one of the aims of the study was to provide guidance on how community members could facilitate help seeking for problem drug users, there was generally low endorsement of directive methods or encouragement for them to seek help. For example, the strategies *The first aider should suggest the person attends a support group, The first aider should offer to make an appointment*, and *The first aider should enlist the help of others (such as a doctor, relative or friend) to confront the person as a group*, were all outright rejected in the first round of the survey. Rejection of the last item is consistent with the research evidence on confrontational approaches (e.g. Johnson Intervention)[20], which highlights a low success rate in engaging the person with treatment services, as well as increasing the risk of long-lasting distress as a result of the perceived betrayal and secrecy involved in their organization [6].

Rather, the guidelines indicate that the general approach the first aider should take is to remain supportive and approachable, support the person in seeking help or changing their behaviour if that is their wish, but not be overly forceful or impinge on their autonomy. Panel members wrote, "*pushing 'seeking help' may interfere with the relationship and cause the drug user stress, which could exacerbate the drug taking*" and "*you can lead a horse to water but you can't make them drink.... you can't force a person to seek professional help, as they will probably continue to use more secretively, which can cause more harm to themselves*". The endorsement that it is the drug user's decision to seek help, and first aiders should not force them to, is consistent with mental health first aid guidelines for other problems (e.g. problem drinking, suicidal thoughts and behaviours). These acknowledge that the role of the first aider is only to support and assist the person if they want to seek help, subject to particular caveats. However, the guidelines acknowledge the difficulty first aiders face in maintaining a good relationship with the person while accepting that they cannot make the person change if they do not want to reduce or cease their problem drug use. One endorsed exception to ensuring the person's autonomy was permission for first aiders to disclose the person's drug use to a professional when they were at risk of harming others.

Some family members or partners engage in enabling behaviours that potentially reinforce the person's ongoing drug use. These behaviours include specific types of caretaking (e.g. taking over childcare or paying living expenses) and attempts to stabilise external situations caused or exacerbated by drug use (e.g. making excuses or lying to others to protect the drug user) [21,22]. These occur as a way of coping with the drug abuse when the person refuses to change their behaviour or seek help. Enabling behaviours are assumed to be maladaptive and are often a target for clinical interventions. Panel members were generally consistent with this view of enabling, with endorsement of most of the items that discouraged enabling behaviours. The strategy *The first aider should not take on the person's responsibilities *was endorsed by all three panels in Round 1, and the strategies *The first aider should not use drugs with the person *and *The first aider should not cover up or make excuses for the person *were accepted in Round 2 after re-rating. However, *The first aider should not provide the person with money to buy drugs *and *The first aider should never get involved with helping the person acquire drugs, e.g. driving the person to meet the dealer *were not endorsed as first aid strategies. Strategies that took a harder line on discouraging enabling were less likely to be endorsed. For example, the strategies *The first aider should deny the person basic needs, such as keeping them warm, clean and nourished*, and *The first aider should hide or throw out the person's drugs *were only endorsed by about 3% of panel members.

The study involved an international sample of experts in problem drug use. These were selected as 'information-rich' sources of expertise. However, it was apparent after the first round of the questionnaire that panel members found it hard to relate to the concept of helping someone with a *developing*, rather than just an *entrenched*, drug use problem, and in situations beyond the more familiar physical health crises requiring physical first aid. Therefore, although a departure from the usual Delphi approach, it was decided to resubmit some strategies to the panel to rate. We emphasized more clearly that the strategies were for developing disorders, as well as entrenched drug problems, in order to make sure that strategies were not being rejected because panel members found them inappropriate for those with entrenched drug disorders alone. However, as noted above, panel members did not significantly change their ratings, indicating that first aid recommendations do not differ depending on the stage of problem drug use.

The guidelines were developed specifically for Western, English speaking countries and may have limited applicability to other countries. They also may not be applicable to cultural minorities within English-speaking countries. However, other mental health first aid guidelines have been adapted for other cultures (e.g. Problem drinking for Australian Aboriginal and Torres Strait Islander Peoples [23]) so adaptations for other cultures are possible. The guidelines were designed to inform the content of MHFA training programs, and have been used in the development of an improved second edition of the MHFA training course [24,25]. Although previous trials have found MHFA training effective in improving knowledge, reducing stigma and increasing helping behaviour [1], studies of the updated training course are required to ensure its effectiveness and that there are not unintended harms, such as labelling people in a way that might increase stigma and marginalization. The use of the guidelines as a stand-alone document is also yet to be tested, but research is currently underway to investigate whether people who download the guidelines from the MHFA website find them useful in providing first aid.

## Conclusions

In conclusion, these guidelines provide best practice mental health first aid strategies for problem use of cannabis, ecstasy, amphetamines, cocaine or heroin. The guidelines will provide an important resource for members of the public seeking to help someone with problem drug use. It is hoped that the guidelines will lead to better support for those with problem drug use and will facilitate earlier intervention. Future research should assess the effectiveness of the first aid strategies endorsed within these guidelines to ensure that they increase supportive behaviour and help during crisis situations.

## Competing interests

Anthony Jorm is research director of MHFA and Claire Kelly is co-ordinator of Youth MHFA.

## Authors' contributions

AJ and DL designed the study and wrote the protocol with input from KH, CK and AK. AK completed the literature review, initial survey construction, recruitment of participants, data collection and analysis, and prepared drafts of the guidelines. A working group, consisting of AJ, DL, AK, KH, CK and LH, gathered regularly to give feedback and make improvements on each draft of the Rounds 1, 2 and 3 surveys and the final guidelines. AM wrote the first draft of the manuscript with input from AJ, AK and DL. All authors have contributed to and approved the final manuscript.

## Pre-publication history

The pre-publication history for this paper can be accessed here:

http://www.biomedcentral.com/1471-244X/11/3/prepub

## Supplementary Material

Additional file 1 **Items that received 80% consensus across the consumer, carer and clinician panels**. Click here for file

Additional file 2 **Helping someone with problem drug use: Mental Health First Aid guidelines**. This file may be distributed freely, with the authorship and copyright details intact. Please do not alter the text or remove the authorship and copyright details. Click here for file
