# Supplementary material for: Helping someone with problem drug use: a delphi consensus study of consumers, carers, and clinicians
Source: BMC Psychiatry. 2011 Jan 5;11:3. doi: 10.1186/1471-244X-11-3 (PMC3022807; doi:10.1186/1471-244X-11-3)
Supplement: Additional file 2 — Helping someone with problem drug use: Mental Health First Aid guidelines. This file may be distributed freely, with the authorship and copyright details intact. Please do not alter the text or remove the authorship and copyright details. [file 1471-244X-11-3-S2.PDF]

MENTAL HEALTH FIRST AID

HELPING SOMEONE WITH PROBLEM DRUG USE

MENTAL HEALTH FIRST AID GUIDELINES

What is problem drug use?

Problem drug use refers to using drugs (e.g. cannabis, ecstasy, amphetamines, cocaine and/or heroin) at levels which are associated with short-term and/or long-term harm (see box "Consequences of problem drug use'). Problem drug use is not just a matter of how much drug the person uses, but how their use affects their life and the lives of those around them. You cannot assume that any use of drugs necessarily means that the person has a drug use problem.

Alcohol is also a drug. If you are concerned that the person may have a drinking problem, please see *Helping someone with problem drinking: mental health first aid guidelines.*

Consequences of problem drug use

You should know the short-term and long-term consequences of problem drug use. These include:

- Adverse effects on the person's judgement and decision-making
- Family or social difficulties (e.g. relationship, work, financial problems)
- Legal problems
- Injuries while using drugs (e.g. as a result of accidents, falls, violence, road trauma)
- Mental health problems (e.g. panic attacks, psychosis, suicidal thoughts and behaviours)
- Physical health problems
- Difficulty controlling the amount of time spent using or the quantity used
- Needing more of the drug to get the same effect
- Problems in cutting down or controlling use
- Experiencing unpleasant symptoms when stopping or reducing use

The MHFA Training & Research Program

Orygen Youth Health Research Centre

The University of Melbourne

AUSTRALIA

www.mhfa.com.au

HELPING SOMEONE WITH PROBLEM DRUG USE

MENTAL HEALTH FIRST AID GUIDELINES

MENTAL HEALTH FIRST AID

Approaching the person about problem drug use

Before speaking to the person, reflect on their situation, organise your thoughts and decide what you want to say. Be aware that the person may react negatively when approached. One of the reasons may be that the person does not consider their drug use a problem. If you are uncertain about how best to approach the person about your concerns, you can speak with a health professional who specialises in problem drug use. You may also find it helpful to consult with others who have dealt with problem drug use about effective ways to help.

Arrange a time to talk with the person. Express your concerns non-judgmentally in a supportive, non-confrontational way. Be assertive, but not do blame or be aggressive. Let the person know that you will listen without judging them (see box 'Tips for effective communication').

Tips for effective communication

- Stay calm and reasonable
- Ask the person about their drug use rather than make assumptions about their use
- When the person finishes talking, repeat back what you have heard them say and allow them to clarify any misunderstandings
- Focus the conversation on the person's behaviour rather than their character
- Use "I" statements instead of "you" statements (e.g. "I feel worried/angry/frustrated when you…" instead of "You make me feel worried/angry/frustrated…")
- Stick to the point (i.e. focus on the person's drug use) and do not get drawn into arguments or discussions about other issues
- Do not criticise the person's drug use
- Do not call the person an "addict" or use other negative labels

Try to talk to the person in a quiet, private environment at a time when there will be no interruptions or distractions and when both of you are in a calm frame of mind. Talk to the person about their drug use by asking about areas of their life it may be affecting (e.g. their mood, work performance and relationships). Ask the person if they would like information about problem drug use or any associated risks. If they agree, provide them with relevant information (e.g. increased risk of physical and mental health problems).

There are a wide range of reasons why people take drugs and the person may not be clear about why they use. Try to find out whether the person wants help to change their drug use. If they do, offer your help and discuss what you are willing and able to do. Have an alcohol and other drug helpline number with you so the person can call for confidential help or ask for more information. Do not expect a dramatic shift in the person's drug use right away; this conversation may be the first time they have thought of their drug use as a problem.

What to do if the person is unwilling to change their drug use

If the person does not want to reduce or stop their drug use you cannot make them change. Do not feel guilty or responsible for their decision to keeping using drugs. It is important that you maintain a good relationship with the person as you may be able to have a beneficial effect on their use. Let the person know you are available to talk in the future.

If the person is unwilling to change their drug use, do not:

- use negative approaches (such as lecturing or making them feel guilty) as these are unlikely to promote change
- try to control them by bribing, nagging, threatening or crying
- use drugs with them
- take on their responsibilities
- cover up or make excuses for them
- deny their basic needs (e.g. food or shelter)

If the person continues to take drugs, you should encourage the person to seek out information (e.g. reputable websites or pamphlets) about ways to reduce risks associated with drug use. If the person is using or planning to use drugs while pregnant or breastfeeding, encourage them to consult with a health professional (e.g. a doctor). You should only disclose the person's drug use to a professional if the person is at risk of harming others.

Professional and other help

There are effective interventions for problem drug use. Treatment options and support services available include education, counselling, therapy, rehabilitation and self-help groups. It is useful to be aware that while abstinence may be a suitable treatment aim for some people, many programs recognise that for others this may not be possible or realistic.

If the person wants professional help

Provide the person with a range of options that they can pursue including information about local services. Encourage the person to find a health professional who they feel comfortable talking to and to make an appointment. Reassure the person that professional help is confidential.

If the person does not want professional help

Be prepared for a negative response when suggesting professional help. It is common for people with problem drug use to initially resist seeking, or to have difficulty accepting, professional help. Drug use is often associated with stigma and discrimination, which are barriers to seeking help.

It is ultimately the person's decision to get professional help. Pressuring the person or using negative approaches may be counter-productive. Be patient and remain optimistic because opportunities will present themselves to suggest professional help again. Changing problem drug use is a process that takes time. Be prepared to talk to the person again in the future. In the meantime, set boundaries around what behaviour you are willing and unwilling to accept from the person.

If the person needs other supports

Encourage the person to talk to someone they trust (for example, a friend, family member or community support worker). Inform the person of supports they may turn to (e.g. self-help resources, support groups, family members) and allow the person to decide which would be most appropriate or useful for them.

HELPING SOMEONE WITH PROBLEM DRUG USE

MENTAL HEALTH FIRST AID GUIDELINES

Drug-affected states

Drug-affected states refer to temporary alterations in the person's mental state or behaviour as a result of drug use, resulting in distress or impairment. The effects of drugs vary from person to person and the behavioural signs of drug-affected states vary depending on the person's level of intoxication. Also, illicit drugs can have unpredictable effects as they are not manufactured in a controlled way. Finally, it is often difficult to make a distinction between the effects of different drugs.

What to do if the person is in a drug-affected state

Stay calm and assess the situation for potential dangers. Try to ensure that the person, yourself and others are safe.

Talk with the person in a respectful manner using simple, clear language. Be prepared to repeat simple requests and instructions as the person may find it difficult to comprehend what has been said. Do not speak in an angry manner.

Try to dissuade the affected person from engaging in dangerous behaviours, such as driving a vehicle or operating machinery. Tell the person that it is dangerous to drive even though they may feel alert.

Encourage the person to tell someone if they start to feel unwell or uneasy, or to call emergency services if they have an adverse reaction.

Adverse reactions leading to a medical emergency

Drug use can lead to a range of medical emergencies. Even though there may be legal implications for the person, it is important that you seek medical help for the person if required and that you tell medical staff that the person has been using drugs.

Adverse physical reactions

You should be able to recognise and help someone who is showing signs of an adverse physical reaction after drug use, such as deteriorating or loss of consciousness, overheating, dehydration and overhydration.

Deteriorating or loss of consciousness

It is a medical emergency if the person shows signs of a rapid deterioration in consciousness (i.e. sudden confusion or disorientation) or unconsciousness (i.e. they fall asleep and cannot be woken). If the person is showing these signs, it is essential that you:

- *Check the person's airway, breathing and circulation*
  You should clear the person's airway if it is blocked. If they are not breathing, give the person expired air resuscitation (EAR). If they don't have a pulse, give the person cardiopulmonary resuscitation (CPR). If you do not know how to give resuscitation (EAR, CPR), enlist the help of someone in the vicinity who knows or call the ambulance service and follow the directions of the telephone operator.
- *Put the person in the recovery position*
  If the person is unconscious, or slipping in and out of consciousness, put them in the recovery position. Ensure they do not roll out of the recovery position onto their back (see box 'Helping an unconscious person').
- *Call an ambulance*
  When you call for an ambulance, it is important that you follow the instructions of the telephone operator. When asked, describe the person's symptoms and explain that the person has been using drugs (e.g. 'my friend has taken a drug, has collapsed and is unconscious'). Try to get detailed information about what drugs the person has taken by asking the person, their friends or visually scanning the environment for clues. Have the address of where you are ready to give to the telephone operator and stay with the person until the ambulance arrives.

Overheating and dehydration

Prolonged dancing in a hot environment (such as a dance party) while on some drugs (e.g. ecstasy) without adequate water intake, can cause the person's body temperature to rise to dangerous levels. This can lead to symptoms of overheating or dehydration, such as:

- feeling hot, exhausted and weak
- persistent headache
- pale, cool, clammy skin
- rapid breathing and shortness of breath
- fatigue, thirst and nausea
- giddiness and feeling faint

If the person is showing symptoms of overheating or dehydration, you must keep the person calm and seek medical help immediately. Encourage the person to stop dancing and to rest somewhere quiet and cool. While waiting for help to arrive, reduce the person's body temperature gradually so as not to induce shock (a life threatening condition brought on by a sudden drop in blood flow throughout the body). Do this by loosening any restrictive clothing or removing any additional layers, and encourage the person to sip non-alcoholic fluids (e.g. water and soft drinks). Prevent the person from drinking too much water at once as this may lead to coma or death. Discourage the person from drinking alcohol as it will further dehydrate them.

Helping an unconscious person

Any unconscious person needs immediate medical attention and their airway kept open. If they are left lying on their back they could suffocate on their vomit or their tongue could block their airway. Putting the person in the recovery position will help to keep the airway open. Before rolling the person in the recovery position, check for sharp objects (e.g. broken glass or syringes) on the ground. If necessary, clear the person's airway after they have vomited. Keep the person warm without allowing them to overheat. Do not inject any substances into the person including salt solution or amphetamine.

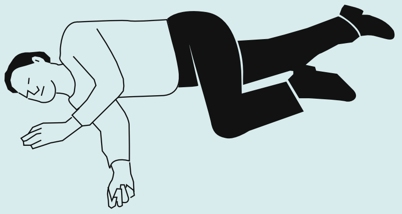

The recovery position

MENTAL HEALTH FIRST AID

HELPING SOMEONE WITH PROBLEM DRUG USE

MENTAL HEALTH FIRST AID GUIDELINES

Adverse psychological reactions

Mental health problems can be caused or exacerbated by drug use. However, it can be difficult to differentiate between the symptoms of mental illness and drug-affected behaviour. You should be able to recognise and help someone who is experiencing an adverse psychological reaction to drugs, such as panic attacks, psychosis, suicidal thoughts and behaviours, and aggression.

Panic attacks

If the person is anxious and panicky, take them to a quiet environment away from crowds, loud noise and bright lights and monitor them in case their psychological state deteriorates. For more information see *Panic attacks: first aid guidelines*.

Psychosis

If the person is experiencing psychosis you should encourage them to seek professional help whether you think the psychosis is drug related or not. For more information see *Psychosis: first aid guidelines*.

Suicidal thoughts or behaviours

For information on helping someone see *Suicidal thoughts and behaviours: first aid guidelines*.

What to do if the person is aggressive

If the person becomes aggressive, assess the risks to yourself, the person and others. Ensure your own safety at all times so that you can continue to be an effective helper. If you feel unsafe, seek help from others. Do not stay with the person if your safety is at risk. Remain as calm as possible and try to de-escalate the situation with the following techniques:

- Talk in a calm, non-confrontational manner
- Speak slowly and confidently with a gentle, caring tone of voice
- Try not to provoke the person; refrain from speaking in a hostile or threatening manner and avoid arguing with them
- Use positive words (such as "stay calm") instead of negative words (such as "don't fight") which may cause the person to overreact
- Consider taking a break from the conversation to allow the person a chance to calm down
- Try to provide the person with a quiet environment away from noise and other distractions
- If inside, try to keep the exits clear so that the person does not feel penned in and you and others can get away easily if needed

If violence has occurred, seek appropriate emergency assistance.

Purpose of these Guidelines

These guidelines are designed to help members of the public provide mental health first aid to someone who may be experiencing problems associated with the use of drugs such as cannabis, ecstasy, amphetamines, cocaine and/or heroin. The first aider's role is to assist the person until appropriate professional help is received or until any drug-related crisis is resolved. The role of a first aider may be filled by any member of the community (e.g. a friend, family member or colleague). The first aider does not necessarily have professional training in drug and alcohol, mental health or medical/emergency care.

Development of these Guidelines

The following guidelines are based on the expert opinions of a panel of consumers, carers and clinicians from Australia, Canada, New Zealand, the USA, and the UK about how to help someone who may have a drug use problem. Details of the methodology can be found in: (citation to follow)

How to use these Guidelines

These guidelines are a general set of recommendations about how you can help someone who may have a drug problem. Each individual is unique and it is important to tailor your support to that person's needs. These recommendations therefore may not be appropriate for every person with problem drug use. Problems with one drug may occur at the same time as problems with other drugs or mental health issues. Also, the guidelines are designed to be suitable for providing first aid in developed English-speaking countries. They may not be suitable for other cultural groups or for countries with different health systems.

Although these guidelines are copyright, they can be freely reproduced for non-profit purposes provided the source is acknowledged. Please cite these guidelines as follows:

Mental Health First Aid Training and Research Program. *Helping someone with problem drug use: mental health first aid guidelines*. Melbourne: Orygen Youth Health Research Centre, University of Melbourne; 2009.

Enquiries should be sent to:

Professor Tony Jorm, Orygen Youth Health Research Centre
Locked Bag 10, Parkville VIC 3052 Australia
email: ajorm@unimelb.edu.au

8252_09OYHKJ

All MHFA guidelines can be downloaded from **www.mhfa.com.au**
